# Social gradients in the receipt of medication for attention-deficit hyperactivity disorder in children and young people in Sheffield

**DOI:** 10.1192/bjo.2019.87

**Published:** 2020-02-07

**Authors:** Samuel P.T. Nunn, Evangelos I. Kritsotakis, Val Harpin, Jack Parker

**Affiliations:** Foundation Year 2 Doctor, St James University Hospital, UK; Honorary Lecturer in Epidemiology & Medical Statistics, School of Health and Related Research, University of Sheffield, UK; and Associate Professor of Biostatistics, School of Medicine, University of Crete, Greece; Consultant Neurodevelopmental Paediatrician, Ryegate Children's Centre, UK; Research Fellow, School of Health and Related Research, University of Sheffield, UK

**Keywords:** Attention deficit hyperactivity disorders, social deprivation, epidemiology

## Abstract

**Background:**

Attention-deficit hyperactivity disorder (ADHD) is a common neurodevelopmental disorder characterised by inattention and hyperactivity-impulsivity that can affect people throughout their life course. A social gradient exists in the prevalence of ADHD in the UK. Studies in other countries have shown that social gradients also exist in the receipt of medication for ADHD. Socioeconomic position is potentially an unrecognised and modifiable factor in children and young people's receipt of medication for ADHD in the UK.

**Aim:**

The aim of the study was to investigate if socioeconomic position could explain in part whether or not children and young people in Sheffield are receiving medication for ADHD.

**Method:**

We used multivariate logistic regression modelling to investigate whether socioeconomic position could explain variation in receipt of medication for ADHD in children and young people in a cross-sectional study. We collected data from 1354 children and young people with a diagnosis of ADHD across three Sheffield centres between January and December 2016. Independent variables were age, gender, religion, ethnicity, comorbidities, and Index of Multiple Deprivation decile (derived from home postcode).

**Results:**

Our results showed a social gradient in the receipt of medication for ADHD (*P*<0.01); an increase in one decile of the Index of Multiple Deprivation was associated with 10% lower odds of receipt of medication for ADHD (adjusted odds ratio 0.90, 95% CI 0.84–0.97).

**Conclusion:**

Children and young people from more deprived backgrounds are more likely to receive medication for ADHD. This is the first time that a social gradient in children and young people's receipt of medication for ADHD has been shown in a UK sample.

Attention-deficit hyperactivity disorder (ADHD) is a neurodevelopmental disorder^1^ characterised by inattention and hyperactivity-impulsivity that affects 3–5% of school-age children.^2^ A diagnosis in childhood requires at least six of nine criteria (five or more in older adolescents and adults) in each of the domains (inattention and hyperactivity-impulsivity).^3^ Unrecognised and therefore untreated ADHD symptoms can have a serious negative impact on the lives of children and young people (CYP), including their education, social development^4,5^ and family relationships, which contribute to secondary difficulties in later life^5^ as symptoms often persist into adulthood.^3^ These difficulties also impact on the community around them. Mainstays of treatment are firstly behavioural interventions (such as parent management classes) and secondly medication.^4^ The causes of ADHD remain a controversial topic^6^ but key ADHD traits have been shown to be over 70% heritable in meta-analysis.^7^ Despite core features of ADHD being biologically determined,^8^ the environment is also important, as this frames the individual and defines how they present and function.^9^ It is likely that social factors affect the presentation of ADHD,^4^ contributing to the heterogeneity of ADHD presentation.

## Social gradients in ADHD

Large UK studies have demonstrated a social gradient in the prevalence of ADHD, finding that those in a lower socioeconomic position (SEP) were more likely to have a diagnosis of ADHD.^10^ Even when a social gradient is found, some contend that this is a covariate of ADHD rather than a cause,^4^ although there is evidence that the effect is causal in severe early deprivation.^11^ Social gradients have also been found in CYP's receipt of medication for ADHD.^12^ In a study of over 2 million CYP in Sweden, the risk of ADHD medication was higher for those aged 10–14 years, those with more frequent hospital contact and those taking other psychotropic medication.^13^ A similar study in British Colombia found that boys are more likely to receive methylphenidate even when controlling for ADHD symptoms and behavioural symptoms, and that prior treatment with methylphenidate was a strong predictor of receipt of treatment.^14^ A US study found that being male, school-aged, White, living in a rural area, and being in foster care increased the likelihood of being treated with psychotropic drugs.^15^ Another national study in Sweden found that having a lone parent, receipt of social welfare and a lower level of maternal education were associated with taking medication for ADHD.^12^ Although several studies worldwide have identified predictors of receiving medication for ADHD, no such research has yet been carried out in the UK. This is an important gap in knowledge, as identification and quantification of a social gradient in the receipt of medication for ADHD in CYP in the UK could make the case for other supportive options to modify this risk factor and improve outcomes in CYP with ADHD. It would provide a rationale for further research into why such a social gradient existed and how it could be alleviated. The objective of our project was to investigate if SEP could explain in part whether or not CYP in Sheffield are receiving medication for ADHD.

## Method

### Study design and setting

The study was based on a cross-sectional sample of all CYP with a recorded diagnosis of ADHD who were seen in three secondary care centres in Sheffield between January and December 2016. Two centres are part of Child and Adolescent Mental Health Services and one is based in a child development centre (Ryegate Children's Centre). All three offer specialist assessment for ADHD. The three centres' case-load includes the majority of CYP with moderate-severe ADHD in Sheffield and the geographical areas included have wide variation in SEP. Sheffield is an ethnically diverse city in South Yorkshire with over 550 000 inhabitants; 36% of Sheffield households include CYP.^16^

Ethical approval was granted by the Research Ethics Committee of the School of Health and Related Research, University of Sheffield.

### Data collection and variables

We collected data on CYP's demographics (age, gender, religion, ethnicity, postcode), comorbid conditions and medication status. Medication status was defined as whether or not CYP were receiving stimulant medication (methylphenidate or dexamphetamine in either immediate or modified-release preparations) or atomoxetine. Comorbidities were classified into autism spectrum disorder (ASD), general or specific intellectual disabilities (also known as learning difficulties in UK health services), or other. ASD and intellectual disabilities were chosen as classifications as these were the two most frequent recorded comorbidities in our sample.

The data were extracted from electronic patient records and clinic letters. We used the Index of Multiple Deprivation (IMD) as a measure of CYP's SEP. The IMD is the official measure of relative deprivation for English lower-level super output layers (areas the size of neighbourhoods); it is a weighted aggregate of data on seven domains of deprivation and ranks neighbourhoods into deciles (the first decile being the most deprived 10% of neighbourhoods).^17^ We used the Department for Communities and Local Government's Open Data Communities postcode lookup service to map to each CYP's home postcode to their IMD decile.^18^

The primary outcome for our analysis was medication status. The main exposure variable was IMD decile. Age, gender, religion, ethnicity and comorbidities were considered as potential confounders.

### Statistical analysis

Analyses were carried out in Stata/IC version 13. Data were presented as mean with standard deviation, or median with interquartile range (IQR), or count with percentage. To detect significant differences between CYP by medication status, numerical data were compared using the Student *t*-test or Mann–Whitney *U*-test depending on the degree of skewness in the data; categorical data were compared using the χ^2^-test.

To evaluate the association between IMD and receipt of medication for ADHD, we used a multivariable logistic regression model controlling for age, gender, ethnicity, religion, ADHD treatment centre and comorbid conditions. The strength and direction of associations were presented using adjusted odds ratios (aORs) with 95% CIs. Multicollinearity among the independent variables was ruled out by examining Spearman's correlations and variance inflation factors. Linearity in the log(odds) for continuous variables (age and IMD), as required by logistic regression, was assessed using restricted cubic splines. We plotted aORs of ADHD medication against the IMD ranks and deciles.

The multivariable logistic regression model was validated by examining its discriminatory and calibration abilities. Calibration (agreement between observed and predicted probabilities of receiving medication for ADHD) was assessed by the Hosmer–Lemeshow goodness-of-fit test and by evaluating how much the slope of the calibration line (plotting the predicted against the observed probabilities) deviates from the ideal of 1.0. Discrimination ability (the degree that the model distinguishes CYP receiving medication from those not receiving medication) was assessed using the area under the receiver operating characteristic curve (c-statistic). Internal validation was performed using the bootstrap method to calculate the c-statistic with correction for ‘optimism’ overfitting based on 200 resamplings. An optimism-corrected c-statistic is reduced by the estimated deterioration that the model is expected to have when applied to new individuals.

Overall, 157/1354 CYP (11.6%) had at least one missing value in any of study variables. ADHD medication status had 113/1354 missing values (8.3%) and IMD had 20/1354 (1.5%). Covariates with missing values included gender (8/1354; 0.6%) and comorbid condition (73/1354; 5.4%). The multivariable logistic regression analysis was performed on a complete case (listwise) basis and the ADHD centre was included in the model as a covariate to reduce potential bias. Potential impact from missing values on the outcome was assessed by performing sensitivity analyses in the two extreme scenarios that: (a) all missing values in medication status were set to ‘medicated’, and (b) all missing values in medication status were set to ‘not medicated’. No imputation was performed on missing covariate values.

## Results

The study sample comprised 1354 CYP diagnosed with ADHD, with a mean age of 13.6 years (s.d. = 3.1, range 5–22 years) and 1113 (82.7%) were males. CYP in the study had a median of 1 comorbid condition (IQR 0–2), including ASD (287 CYP; 22.4%), intellectual disabilities (134 CYP; 10.5%) and ‘other’ comorbidities (509 CYP; 39.7%). Some comorbid conditions, for example oppositional defiant disorder, conduct disorder and developmental coordination disorder were not possible to ascertain fully using our data-collection method and it is likely that their prevalence is significantly higher than figures suggested. Most CYP were of White British ethnic origin (1080; 79.8%), and declared a Christian orientation (527; 38.9%) or ‘no religion’ (662; 48.9%). The median IMD rank was 4.7 (IQR 1.4–15.7; range 0.05–32.82) thousand units, corresponding to a median IMD decile of 2 (IQR 1–5; range 1–10).

Information on medication status was available for 1241 (91.7%) CYP, of whom 1135 were prescribed ADHD medication (91.5%, 95% CI 89.8–92.9%). Table 1 shows the characteristics of the CYP by medication status. Those CYP receiving medication for ADHD were older (mean 13.7 *v.* 13.0 years, *P* = 0.016), more likely to have ASD (23.2% *v.* 14.9%, *P* = 0.055) and had more comorbid conditions (median 1.0 *v.* 0.0, *P* = 0.019) compared with those who did not receive medication for ADHD. The median IMD ranks and deciles were significantly lower in CYP receiving medication for ADHD compared with those not receiving medication (ranks: 4.3 *v.* 8.1 thousands, *P* = 0.017; deciles: 2.0 *v.* 3.0, *P* = 0.021). No significant differences were observed in the distributions of gender, ADHD care centre, ethnic group or religion by medication status.

**Table 1:** Characteristics of patients with attention-deficit hyperactivity disorder (ADHD) in relation to their medication status

Variable	ADHD medication status			*P*^a^
	Receiving medication (*n* = 1135)	Not receiving medication (*n* = 106)	Unknown (*n* = 113)	
Age in years, mean (s.d.)	13.7 (3.0)	13.0 (3.3)	12.7 (3.4)	0.016
Male, *n* (%)^b^	935 (82.7)	85 (81.0)	93 (83.8)	0.643
Care centre, *n* (%)				
Ryegate Children's Centre	771 (67.9)	81 (76.4)	113 (100.0)	0.195
CAMHS 1	139 (12.2)	10 (9.4)	0 (0.0)	
CAMHS 2	225 (19.8)	15 (14.2)	0 (0.0)	
Ethnic group, *n* (%)				
Other	172 (15.2)	22 (20.8)	14 (12.4)	0.293
White British	907 (79.9)	80 (75.5)	93 (82.3)	
Not available	56 (4.9)	4 (3.8)	6 (5.3)	
Religion, *n* (%)				
No religion	549 (48.4)	52 (49.1)	61 (54.0)	0.889
Christian	453 (39.9)	42 (39.6)	32 (28.3)	
Other religion	51 (4.5)	6 (5.7)	11 (9.7)	
Not available	82 (7.2)	6 (5.7)	9 (8.0)	
Comorbid conditions^b^				
Median number (IQR)	1.0 (0.0–2.0)	0.0 (0.0–1.0)	1.0 (0.0–2.0)	0.019
Autism spectrum disorder, *n* (%)	257 (23.2)	15 (14.9)	15 (20.8)	0.055
Intellectual difficulties, *n* (%)	115 (10.4)	7 (6.9)	12 (16.7)	0.271
Other comorbidity, *n* (%)	445 (40.2)	35 (34.7)	29 (40.3)	0.279
Index of multiple deprivation				
Rank × 1000, median (IQR)	4.3 (1.4–14.8)	8.1 (1.7–20.7)	3.7 (1.4–15.7)	0.017
Decile, median (IQR)	2.0 (1.0–5.0)	3.0 (1.0–7.0)	2.0 (1.0–5.0)	0.021

CAMHS, Child and Adolescent Mental Health Services; IQR, interquartile range.

a. Comparing those receiving medication with those not receiving medication (complete case analysis).

b. Percentages were calculated based on the number of individuals with known information. There were 8 missing values for gender and 73 missing values for comorbid condition.

**Fig. 1:**
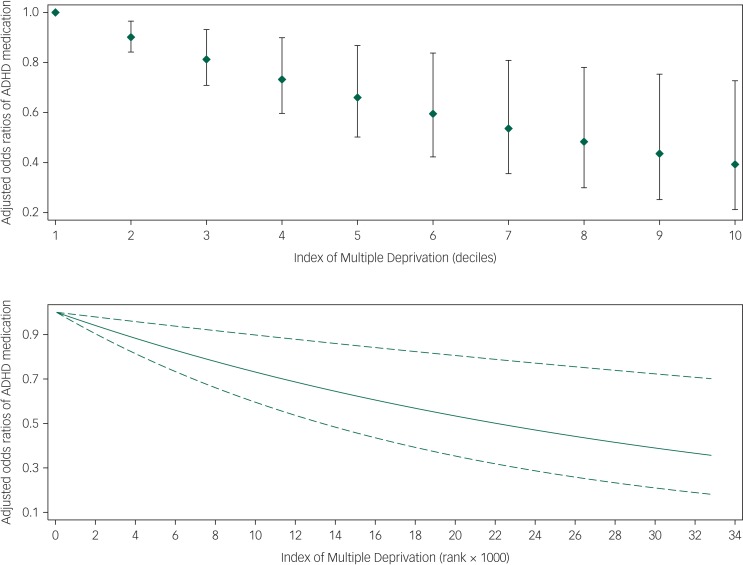
Adjusted odds ratios (diamonds in upper panel; solid lines in lower panel) with 95% confidence interval (capped spikes in upper panel; dashed lined in lower panel) for the relation of the Index of Multiple Deprivation and receipt of medication in children and young adults with Attention Deficit Hyper Activity Disorder (ADHD).

A linear relationship with the log odds of receiving ADHD medication was found to be a good approximation for IMD ranks or deciles, but a non-linear relationship was noted for age, which was modelled using a restricted cubic spline with knots at quartiles (11.3, 13.6 and 16 years) (supplementary Fig. 1 available at https://doi.org/10.1192/bjo.2019.87). Multivariable logistic regression confirmed that the CYP's receipt of medication for ADHD was significantly and independently associated with age, comorbid ASD and IMD decile (Table 2). In particular, an increase in every one decile of IMD (i.e. less deprivation) was associated with a decrease in the odds of receiving ADHD medication by 10% (aOR = 0.90, 95% CI 0.84–0.97) as shown in Fig. 1. The model's discriminative ability was adequate (c-statistic 0.66, 95% CI 0.60–0.72) and internal validation indicated a small degree of overfitting (bootstrap optimism-corrected c-statistic, 0.63). Model predicted probabilities ranged between 55.2% and 98.5%. The Hosmer–Lemeshow test (*P* = 0.370) and the calibration slope (0.91, 95% CI 0.50–1.32) indicated good agreement between predicted and observed probabilities of receiving medication for ADHD. In addition, the calibration plot did not indicate a pattern of either over- or underestimation (supplementary Fig. 2).

**Table 2:** Multivariable logistic regression model for the association between receipt of medication for attention-deficit hyperactivity disorder and the Index of Multiple Deprivation, controlling for patient's age, gender, care centre, ethnic group, religion and comorbid conditions (*n* = 1197)

Variable	Effect measure	*P*
IMD decile, per decile increase, aOR (95% CI)	0.90 (0.84–0.97)	0.003
Age, per year increase, aOR (95% CI)		
Age spline 1	1.19 (1.04–1.36)	0.012
Age spline 2	0.85 (0.72–1.01)	0.066
Female gender, aOR (95% CI)	0.85 (0.50–1.44)	0.537
Care centre, aOR (95% CI)		
Ryegate Children's Centre	1.00	−
CAMHS 1	1.61 (0.77–3.37)	0.208
CAMHS 2	1.63 (0.89–3.00)	0.115
Ethnic group, aOR (95% CI)		
Other	1.00	−
White British	1.77 (0.98–3.20)	0.057
Not stated	2.04 (0.38–10.83)	0.405
Religion, aOR (95% CI)		
No religion	1.00	
Christian	1.00 (0.63–1.58)	0.999
Other religion	1.19 (0.42–3.35)	0.738
Not stated	2.00 (0.49–8.07)	0.332
Comorbid conditions, aOR (95% CI)		
Autism spectrum disorder	1.79 (1.00–3.18)	0.050
Intellectual difficulties	1.33 (0.59 – 3.00)	0.494
Other comorbidity	1.41 (0.90–2.21)	0.139
Model performance statistics		
Calibration slope (95% CI)	0.91 (0.50–1.32)	
HL goodness-of-fit, χ^2^ (d.f.)	8.68 (8)	0.370
C-statistic (95% CI)	0.66 (0.60–0.72)	
C-statistic, BOC	0.63	

IMD, Index of Multiple Deprivation; aOR, adjusted odds ratio; CAMHS, Child and Adolescent Mental Health Services; HL, Hosmer–Lemeshow; d.f., degrees of freedom; BOC, bootstrap optimism corrected. Age was modelled using restricted cubic splines with slopes defined at quartiles (11.3, 13.6 and 16.0 years).

Odds ratios were estimated using logistic regression adjusting for age, sex, ethnicity, religion, care centre and comorbid conditions (*n* = 1197).

Sensitivity analysis accounting for potential bias from missing values in medication status produced identical results (supplementary Table 1). The aORs of receiving ADHD medication in relation to IMD decile were: 0.90 (95% CI, 0.85–0.97; *P* = 0.004) assuming CYP with missing data were receiving medication for ADHD, and 0.94 (95% CI, 0.89–0.99; *P* = 0.024) assuming those CYP with missing data were not receiving medication for ADHD.

## Discussion

Our results showed a social gradient in the receipt of medication following a diagnosis of ADHD. CYP from a more deprived background were more likely to be receiving medication for their ADHD, controlling for age, gender, ethnicity, religion and comorbid disorders. CYP's receipt of medication for ADHD was significantly and independently associated with increasing age and comorbid ASD.

### Interpretation

Our data showed that an increase in one decile of IMD (i.e. less deprivation) was associated with 10% lower odds of receipt of medication for ADHD (aOR = 0.90; 95% CI, 0.84–0.97). The finding that CYP from more deprived backgrounds are more likely to be receiving medication has been observed in similar studies in Sweden^12^ and Denmark,^19^ but has not been demonstrated in the UK before. It is unlikely that the relationship between SEP and receipt of medication for ADHD is monofactorial; and to posit that would probably be a gross oversimplification of a complex (and possibly bi-directional) relationship with many interacting factors, one of which we have shown is comorbid ASD. However, the existence of a social gradient in CYP's medication status is important because ADHD is a common disorder that can lead to serious negative outcomes for CYP.

### Mechanisms of the social gradient

It is known that social setting influences the presentation of childhood/adolescent ADHD^4^ and that deprivation makes it more likely that CYP are exposed to a negative family, school and community environment known to worsen ADHD presentation.^20^ Secondary analysis of the Millennium Cohort Study data found that family dynamics mediated the relationship between SEP and ADHD symptoms,^21^ and it may also be the case that the same applies to receipt of medication for ADHD. In this study we do not have a measure of severity of ADHD. The social gradient in receipt of medication for ADHD could be because CYP from deprived backgrounds present with more severe impairment and so are more likely to be receiving medication. It is possible to postulate many reasons for this. Families living in more deprived circumstances may be less likely or even less able to seek help for possible ADHD. If they do seek help, professionals, including teachers (who often instigate referrals for possible ADHD) and general practitioners, may be more likely to attribute the reported difficulties to social circumstances and poor parenting rather than ADHD.

It is important to consider which CYP are referred for secondary assessment of possible ADHD in Sheffield and therefore constitute our sample – a specialist referral is indicated if moderate/severe impairment is thought to be present. Although it is hoped that CYP with significant mental health comorbidity will be referred to Child and Adolescent Mental Health Services, and those with other neurodevelopmental difficulties to the child development centre, the nature of ADHD is such that CYP often have additional difficulties in both areas, thus all three centres see individuals with complex cases. We did not identify differences in whether CYP with ADHD were receiving medication between centres of care, however, it is possible that there are some differences in practices because of the individual clinicians working in each team.

Access to services has also been shown to be a barrier to ADHD parenting group attendance, along with dislike of group activities, preconceptions of parenting programmes, inability to engage with the programme (lack of understanding or finding the programme unhelpful), or changes in circumstances.^22^ Each of these factors are more likely in a deprived household where carers are likely to have lower educational achievement or additional work and family stressors. Not being able to benefit from first-line therapy for ADHD (such as parent management classes and other non-pharmacological treatments) will lead to an increased chance of receiving pharmacological treatment as a second-line therapy. It is also likely that families in poorer socioeconomic circumstances do not have the finances needed to offer their CYP's additional support that can reduce impairment from ADHD and therefore the need for medication, such as sporting activities, additional educational support and more individual adult support.

### ADHD may affect SEP

It is also possible that ADHD has a part to play in determining an individual's SEP, which could explain in part why those with a lower SEP are more likely to be receiving medication. It is well-known that unrecognised and therefore untreated ADHD leads to a range of negative outcomes including poor educational attainment, fewer occupational opportunities, alcohol and substance misuse, contact with the criminal justice system, and poor mental health.^5^ Thus, adults with ADHD are more likely to live in deprived circumstances. As ADHD traits are highly heritable,^7^ this effect may have been compounded over many generations, which would lead to more severe impairment from ADHD, and therefore leading to CYP in such a position being more likely to be receiving medication. One previous study did not find any evidence that families with CYP with ADHD were more likely to move to a lower SEP;^21^ although the study was only over a period of 7 years and may not have run for long enough to detect changes in SEP.

### Secondary findings

The clinical significance of our secondary finding that CYP with comorbid ASD are more likely to receive medication for ADHD is unclear. This subgroup may also be more likely to be receiving medication as they have a higher level of impairment,^23^ and should be considered at particular risk of negative outcomes because of multiple layers of vulnerability.

The simplest explanation of our finding that CYP who are older are more likely to be receiving medication is that the 2008 National Institute for Health and Care Excellence (NICE) guidance recommended medication as the second-line treatment for all but the most severe ADHD and that conservative measures should be tried first.^4^ The review of NICE guidance in March 2018 recommends medication first line for those with moderate and severe ADHD so this finding may change over time.^24^

### Limitations

A key limitation in this study is the cross-sectional design – a lack of temporality makes it difficult to infer that SEP is a causal factor of ADHD medication status from our study alone. Severity of ADHD was not recorded and should be included in future work; similarly, educational attainment may be relevant for further work. Our measures of ethnicity could have been more discriminate, which would be important to consider in further work as ethnicity has been shown to be a predictor of whether children are receiving medication. Our study cannot investigate further why such a social gradient exists, which is important to consider when evaluating whether SEP is a modifiable risk factor. More population-based data collection over time is needed. Although our cohort contained a large proportion of CYP with ADHD in Sheffield, it is possible that there was some selection bias in the cohort that cannot be accounted for in the study.

### Generalisability

Sheffield is a diverse city in terms of ethnicity, income and religion; our sample was a large cross section across these demographic facets, with our study cohort making up a substantial proportion of all CYP with ADHD in Sheffield. These factors make it likely that the findings of this study are generalisable to other cities in the UK.

### Implications for practice

The social gradient in CYP's receipt of medication represents a novel risk factor that clinicians should aim to modify. Because a social gradient in the receipt of medication for ADHD exists, those from more deprived backgrounds should be considered especially vulnerable regardless of causality, with clinicians thinking of social deprivation in the same way as an additional comorbidity. Targeted interventions for those from a lower SEP may be effective, but progress may be difficult unless the wider inequality in our society is addressed. Further research into why the social gradient in CYP's receipt of medication exists would be helpful to determine if interventions are appropriate; and studies of the long-term effects of increased medication use may also aid service development.

This social gradient presents a challenge not only to clinicians but even more to politicians, public health professionals and social care, and adds further weight to the argument that tackling the social determinants of health should be a priority for public health professionals and policymakers alike.

In conclusion, a social gradient is evident in the prevalence of ADHD and in CYP's receipt of medication in the UK. To the best of our knowledge, this is the first study that has shown a social gradient in ADHD drug treatment outcomes in a UK population.

## Data Availability

S.P.T.N., E.I.K. and J.P. each had access to the full study data-set.

## Supplementary material

For supplementary material accompanying this paper visit http://dx.doi.org/10.1192/bjo.2019.87. click here to view supplementary material
