# Supplementary material for: Social gradients in the receipt of medication for attention-deficit hyperactivity disorder in children and young people in Sheffield
Source: BJPsych Open. 2020 Feb 7;6(2):e14. doi: 10.1192/bjo.2019.87 (PMC7176834; doi:10.1192/bjo.2019.87)
Supplement: Supplementary file 1 [file S2056472419000875sup001.zip › S2056472419000875sup001/NunnS_et_al_Table_3_(supplementary).docx]

**Supplementary Table 3**. Sensitivity analyses to assess the impact of missing values on medication status. Results of logistic regression models of the odds of receiving ADHD medication with unknown ADHD medication status set to ‘medicated’ (Model 1; n=1268), and unknown ADHD medication status set to ‘not medicated’ (Model 2, n=1268).

| **Variable** | **Model 1** | | | **Model 2** | | |
| --- | --- | --- | --- | --- | --- | --- |
|  | **aOR** | **95%CI** | **P value** | **aOR** | **95%CI** | **P value** |
| IMD decile, per decile increase | 0.90 | 0.85 – 0.97 | 0.004 | 0.94 | 0.89 – 0.99 | 0.024 |
| Age, per year increase |  |  |  |  |  |  |
| Age spline 1 | 1.17 | 1.02 - 1.33 | 0.022 | 1.20 | 1.08 - 1.33 | 0.001 |
| Age spline 2 | 0.86 | 0.73 - 1.02 | 0.088 | 0.85 | 0.75 - 0.98 | 0.021 |
| Female sex | 0.83 | 0.49 - 1.41 | 0.499 | 0.99 | 0.64 - 1.53 | 0.963 |
| Care Centre |  | - |  |  | - |  |
| RCC | 1.00 | - |  | 1.00 | - | - |
| C-CAMHS | 1.47 | 0.70 - 3.08 | 0.305 | 2.82 | 1.38 - 5.76 | 0.004 |
| B-CAMHS | 1.49 | 0.81 - 2.74 | 0.198 | 3.01 | 1.69 - 5.35 | <0.001 |
| Ethnic group |  | - |  |  | - |  |
| Other | 1.00 | - |  | 1.00 | - | - |
| White British | 1.78 | 0.99 - 3.21 | 0.056 | 1.54 | 0.92 - 2.56 | 0.100 |
| Not stated | 1.90 | 0.36 - 10.09 | 0.452 | 1.88 | 0.57 - 6.22 | 0.304 |
| Religion |  | - |  |  | - |  |
| No religion | 1.00 | - |  | 1.00 | - | - |
| Christian | 0.98 | 0.62 - 1.54 | 0.922 | 1.18 | 0.82 - 1.71 | 0.375 |
| Other religion | 1.25 | 0.45 - 3.48 | 0.674 | 1.07 | 0.48 - 2.41 | 0.865 |
| Not stated | 2.07 | 0.51 - 8.38 | 0.307 | 1.44 | 0.55 - 3.75 | 0.459 |
| Comorbid conditions |  | - |  |  | - |  |
| Autism spectrum disorder | 1.74 | 0.98 - 3.09 | 0.061 | 1.42 | 0.92 - 2.18 | 0.112 |
| Learning difficulties | 1.37 | 0.61 - 3.07 | 0.448 | 0.83 | 0.48 - 1.42 | 0.491 |
| Other comorbidity | 1.38 | 0.88 - 2.16 | 0.161 | 1.40 | 0.98 - 1.99 | 0.065 |
| **Model performance statistics** |  |  |  |  |  |  |
| HL goodness of fit, chi-square (df) | 8.15 (8) |  | 0.419 | 12.05 (8) |  | 0.149 |
| C-statistic | 0.65 | 0.60 – 0.71 |  | 0.67 | 0.63 – 0.71 |  |

IMD, Index of Multiple Deprivation; aOR, adjusted odds ratio; CI, confidence interval; CAMHS, Child and Adolescent Mental Health Services; BOC, bootstrap optimism corrected; df, degrees of freedom; HL, Hosmer-Lemeshow.
